# Determining the Causes of Mental Health Issues in Airline Pilots: The Role of Working Conditions and Workload in Predictive Models

**DOI:** 10.7759/cureus.103870

**Published:** 2026-02-18

**Authors:** Emanuel Schad, Teresa Knueppel, Timo-Kolja Pfoertner

**Affiliations:** 1 Medical Sociology, University of Cologne, Cologne, DEU; 2 Forensic Medicine, University of Cologne, Cologne, DEU; 3 Faculty of Human Sciences, University of Cologne, Cologne, DEU

**Keywords:** airline pilots, depression, flight safety, mental health, suicide, working conditions, workload

## Abstract

The mental health of airline pilots is critical to the safety of air travel. This study aimed to investigate the prevalence of depressive disorders among airline pilots and the influence of various work-related factors on their mental health. We conducted a web-based survey from January 2022 to January 2023, collecting data from 277 airline pilots using the Patient Health Questionnaire-9 (PHQ-9) to screen for depressive disorders. Our findings reveal that 29 pilots (10.4%) showed signs of major depressive disorders, and nine pilots (3.2%) reported suicidal thoughts during the past two weeks. We built linear regression models in which psychosocial working conditions and indoor ambient conditions were significant predictors of the PHQ-9 score. Gender, age, flight hours, additional duties, and self-rated performance were not significantly associated with the PHQ-9 score. The study highlights the need for improved working conditions, including job security, a balanced workload, and opportunities to influence working conditions. Mental health support programs could lower the threshold for seeking medical help, thus improving flight safety.

## Introduction

Depressive disorders are one of the emerging global health problems, with over 300 million people living with depression worldwide. For 2015, the World Health Organization (WHO) published an estimated global total of over 50 million years lived with disability (YLD), which equals 7.5% of all YLD and is ranked as one of the largest contributors to all YLD. The report stated an average prevalence of depressive disorders of 4.4% among all countries, with a range between 2.9% and 5.9% depending on the region [1].

When looking for causes and contributing factors for depressive disorders, studies consistently found factors like female sex and unemployment as positively linked to a higher prevalence, whereas a higher income was found as a contributing factor to a lower prevalence of major depressive disorders [2,3]. In contrast, employees in professions with above-average income, such as physicians, have been shown to exhibit a higher prevalence of depressive disorders compared with the general population [4,5].

Another profession with an above-average income is that of airline pilots. A recent study with a sample of over 3,000 airline pilots found an overall prevalence of depressive disorders at 12.6%; in the group of pilots who were actually working as airline pilots during the last 30 days, the percentage was even higher (13.6%) [6]. Other studies found varying prevalence rates of depressive symptoms, ranging from 1.9% to 12.6% [7]. The increased prevalence of depressive disorders among airline pilots may be attributed to their unique working and environmental conditions. For example, a study of Spanish airline pilots investigated the prevalence of fatigue, work overload, and sleepiness in a sample of 283 airline pilots and found elevated levels of these symptoms. This seems plausible in the context of a pilot’s work environment with frequent circadian disruptions and sleep deprivation, and may ease the path into mental disorders like burnout or depression [8].

However, no study has yet established a direct link between specific burdensome working and environmental factors and their association with the mental health of airline pilots. Our study aims to investigate the contributing factors to reduced mental health, specifically depressive disorders, among airline pilots. We seek to elucidate how various working and environmental factors are associated with the prevalence of depressive disorders and how the design of these conditions may influence the occurrence of depressive disorders. Evaluating these factors is of particular interest, as depressive disorders are directly linked to air transport safety, as tragically demonstrated by the Germanwings incident [7]. Additionally, pilots face unique challenges when seeking help for depressive symptoms. In most regions worldwide, their yearly-renewed medical certificates, which allow them to exercise the privileges of their Airline Transport Pilot License (ATPL), are suspended if signs of a psychiatric disorder are detected [9]. This might lead to a possible loss of the job, since most of the airline pilots' working contracts require a valid Class 1 medical certificate as a prerequisite. As the aeromedical examination system relies on self-disclosure in the field of psychiatric disorders [9], it seems imaginable that some airline pilots suffering from depressive symptoms will not seek adequate medical help in order to avoid job loss and a possible social decline. In this context, it seems necessary to evaluate the underlying causes of the above-mentioned elevated level of depressive disorders among airline pilots [6].

## Materials and methods

This was a survey-based study. The study was approved by the ethical committee of the University of Cologne and registered on the WHO clinical trials platform (DRKS00028955). Participation was completely anonymous since we were collecting highly sensitive data.

Participants

The inclusion criteria were: commercial airline pilot with at least one valid type rating, aged 18-65 years. Pilots were recruited with the help of pilot unions (Germany, Austria, Netherlands), via handing out flyers at the Lufthansa Aviation Training campus and German aeromedical examiner offices, via targeted social media groups by advertising on Instagram and Facebook (Meta Platforms, Inc., Menlo Park, California, United States) using a sharply targeted audience and by placing an advertisement on a website collecting flight incidents and accidents (avherald.com).

Data collection

A web-based questionnaire was developed using a customized survey from the LimeSurvey tool (LimeSurvey GmbH, Hamburg, Germany). Data collection was done between January 2022 and May 2023. The survey took approximately 30 minutes to complete. In order to facilitate participation, mandatory questions were not included, and the survey could be exited at any stage.

Participants were asked to complete the questionnaire covering the following aspects: demographic data, current aircraft type, second aircraft type (if applicable), flight hours (total, last year, last month, last five years in average), depression screening using Patient Health Questionnaire-9 (PHQ-9) scores, assessment of working conditions including psychosocial factors, environmental factors and rating of air quality, additional duties, workplace security (COVID-19 impact), fume events, rating of own performance as a pilot, physical symptoms, diagnosed cancer, smoking.

Sample size

**Figure 1:**
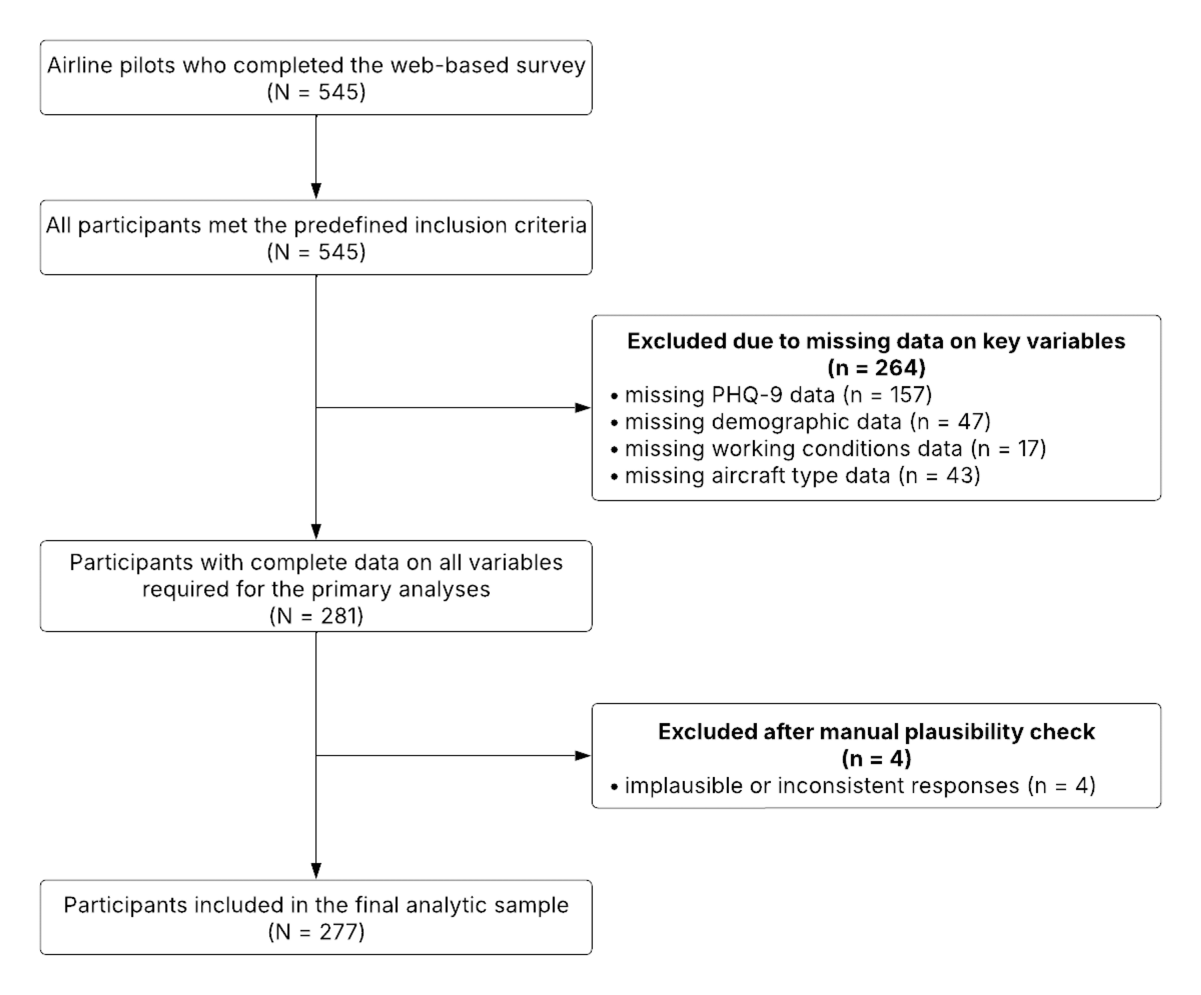
Flow chart illustrating participant recruitment, exclusions, and inclusion in the final analytic sample. A total of 545 airline pilots completed the web-based survey. All participants met the predefined inclusion criteria. Exclusions were based solely on missing data on key variables or implausible responses identified during manual plausibility checks. After exclusion of incomplete datasets and implausible responses, 277 pilots were included in the final analyses. PHQ-9: Patient Health Questionnaire-9

A total of 545 airline pilots completed the web-based survey. Participants were excluded stepwise if information on key variables required for the analyses was missing. First, datasets with incomplete PHQ-9 data were excluded. Subsequently, participants with missing information on aircraft type, country, gender, or working condition variables were removed. All remaining datasets were then manually screened for plausibility, and implausible responses were excluded. The final analytical sample consisted of 277 airline pilots. The participant selection process is illustrated in Figure 1.

Variables

Dependent Variable

To screen for depressive disorders, we used the PHQ-9, which was originally designed for depression screening in hospital patients [10]. The main reasons for our decision were the high level of sensitivity (88%) and specificity (85%) [11], both for a score of 10 or more, and to allow an easier comparability of our data to the data of other studies [6]. The PHQ-9 uses nine questions to assess the occurrence and frequency of depressive symptoms during the last two weeks. Answers range from “not at all”, “several days”, to “more than half the days” and “nearly every day” and are assigned a score rating of zero to three. When assessing the total score, all scores were added, which gave a value between zero and 27. A total score of 10 or more is defined as a cutoff for at least moderate depressive disorders with the above-mentioned high sensitivity and specificity. A mild depressive disorder could be indicated by a total score of five or more [10], but it seems less reliable, with a specificity of only 55% [11]. The PHQ-9 score was used as a metric variable and also as a categorical variable when grouped according to levels of depression.

Independent Variables

To assess different dimensions of working conditions, environmental and psychosocial factors were measured using a set of self-report items addressing psychosocial stressors, environmental conditions, and perceived air quality on board. The responses were recorded using Likert-type scales, with higher scores indicating greater perceived burden. Selected items assessing psychosocial work environment and environmental factors were adapted to the aviation context from the MM-40 questionnaire, reproduced from Lahtinen et al. [12] with permission from BMJ Publishing Group Ltd.

The environmental factors scale consisted of 12 items covering different aspects of the physical working environment. Participants were asked to rate how frequently they had been bothered by each factor during the previous three months. Response options were modified to suit the study population and included “Every flight”, “Majority of the flights”, “At some flights”, and “Never”. These responses were scored from zero to three points, respectively. The assessed factors included draught (flowing air), ambient temperature being too high, too low, or varying, stuffy or “bad” air, dry air, unpleasant odour, static electricity causing shocks, passive smoking, noise, dim or reflective lighting, and dust or dirt. A total score ranging from zero to 33 was calculated and used as a continuous variable in the linear regression analyses.

For the assessment of psychosocial factors, we used five questions from the same publication examining the psychosocial work environment in office workplaces experiencing indoor air problems [12]. Possible answers ranged from “Yes, often”, “Yes, sometimes”, to “No, seldom” and “No, never”. Each answer was assigned a score value of zero to three, where three points were assigned to the negative answer. The total score ranked between zero and 15. We have used this score as a metric variable.

We performed a factor analysis of all the above-mentioned questions via ScreePlot [13], where we could clearly distinguish between two factors (environmental factors and psychosocial conditions). The question concerning passive smoke was eliminated due to a very low factor load of 0.21. In the subsequent reliability analysis, we calculated Cronbach's alpha values of 0.9 for environmental factors and 0.6 for the assessment of working conditions, which was considered as sufficient [14].

The rating of air quality on board was assessed by offering five possible answers ranging from one point “very good” to five points “very bad”, so a total score value of five points was possible. Again, we used this variable as a metric variable.

Control Variables

We further considered age (continuous variable), gender (male, female), flight hours, job security, additional duties, and pilots’ performance as control variables. We used flight hours during the last 12 months as an indicator of the current workload. This variable was used as a metric variable. To take into account the major impact of the COVID pandemic on the aviation industry, we assessed the current job security with four possible answers: “Job safe”, “Job lost”, “Job lost, but found a new job”, and “Job endangered”. Individuals who reported having a secure job or having found a new job after job loss were operationalized as having a secure working place (with the value zero), while those who had lost their job or perceived their job as endangered were operationalized as having no secure working place (with the value one). Pilots’ self-rated performance was assessed using three core competencies (“Aeroplane Flight Path Management, manual control”, “Situational Awareness and Management of Information”, and “Workload Management”) derived from the International Civil Aviation Organization (ICAO) competency-based training and assessment (CBTA) framework [15]. Participants rated their performance on a 10-point Likert scale. The assessment tool was developed by the authors for the purpose of this study and was not based on an official ICAO questionnaire. We calculated a total score by adding up every single score to a maximum of 30 and a minimum of three points. This variable was used as a metric variable.

Statistical analyses

Statistical analysis was done using RStudio version 2023.06.1+524 (Posit PBC, Boston, Massachusetts, United States) and IBM SPSS Statistics for Mac, version 29.0.1.0 (IBM Corp., Armonk, New York, United States). 

After excluding data not matching the inclusion criteria, we manually screened the data for non-plausible datasets, which were deleted. For our descriptive statistical analyses, we used the parameters number of participants (N), number in different groups (n), and the distribution in percentage. For metric variables, we calculated the mean (SD) values. After testing for normal distribution, we used the mean and t-tests or ANOVAs with Bonferroni corrections to identify significant differences. Statistical significance was defined as p<.050.

As a second step, datasets were divided by the PHQ-9 score (<10 points and ≥10 points as an indicator for a minimum moderate depression) and tested for significant differences in every single variable.

As a final step, three different linear regression models were built using the PHQ-9 score as the dependent variable, and the independent variables were gradually introduced, starting with demographic data, adding workload, job security, and additional duties, and finally, the different dimensions of working conditions and pilots’ performance as possible predictors. The variable “Flight hours 12 months” was additionally used as a polynomial factor to test for the effect of low or high workload on the PHQ-9 score.

## Results

In our sample of 277 participants, the mean age was 40.26 ± 10.22 years. Most participants identified as male (n=262, 94.6%), while 15 pilots (5.4%) identified as female (Table 1). The mean number of flight hours recorded over the past 12 months was 374.59 ± 239.89. The majority of pilots reported not fulfilling additional duties (n=197; 71.1%), and 169 pilots (61.0%) indicated having a secure job.

**Table 1:** Categorical characteristics of the study population (N=277) PHQ-9: Patient Health Questionnaire-9

Variable	Overall, n (%)	PHQ-9 score <10, n (%)	PHQ-9 score ≥10, n (%)	p-value
Gender - male	262 (94.6)	235 (94.8)	27 (93.1)	.709
Gender - female	15 (5.4)	13 (5.2)	2 (6.9)	-
Additional duties - yes	80 (28.9)	72 (29.0)	8 (27.6)	.871
Job security - secure	169 (61.0)	156 (62.9)	13 (44.8)	.059

Participants had a mean score of 23.65 ± 3.48 when assessing pilots' performance. Regarding working conditions across the three dimensions evaluated, pilots reported a mean score of 6.31 ± 2.43 for psychosocial conditions, 9.68 ± 5.38 for environmental conditions, and 2.16 ± .86 for air quality on board (Table 2). 

**Table 2:** Continuous characteristics of the study population (N=277) PHQ-9: Patient Health Questionnaire-9

Variable	Overall, mean ± SD	PHQ-9 score <10, mean ± SD	PHQ-9 score ≥10, mean ± SD	p-value
Age	40.26 ± 10.22	40.25 ± 10.38	40.38 ± 8.91	.960
PHQ-9 Score	4.31 ± 4.19	3.21 ± 2.64	13.69 ± 3.18	< .001
Flight hours 12 months	374.59 ± 239.89	366.95 ± 237.47	439.93 ± 254.67	.151
Working conditions - Psychosocial factors	6.31 ± 2.43	6.01 ± 2.27	8.86 ± 2.28	< .001
Working conditions - Environmental factors	9.68 ± 5.38	9.27 ± 5.16	13.17 ± 5.99	< .001
Working conditions - Air quality	2.16 ± .86	2.13 ± .87	2.34 ± 1.05	.301
Pilots’ performance	23.65 ± 3.48	23.81 ± 3.51	22.24 ± 2.96	.012

Mean PHQ-9 score was 4.31 ± 4.19. Twenty-nine participants (10.4%) had a score value that could be indicative of at least a moderate depressive disorder, of which 17 (6.1%) showed a score value for moderate, 11 (4.0%) for moderately severe, and one (0.4%) for severe depression (Tables 2, 3). Moreover, nine participants (3.2%) indicated having suicidal thoughts during the last two weeks (Table 3). Results indicated significant differences in the presence of a score value that is indicative of at least moderate depressive disorder (PHQ-9 ≥10) in the psychosocial and environmental factors, and pilot performance (Table 2).

**Table 3:** PHQ-9 score, depression severity, and suicidal thoughts PHQ-9: Patient Health Questionnaire-9

Variable	Frequency	Percentage	PHQ-9 score, mean ± SD	Depression Severity
PHQ-9 Score 0 – 4	174	62.8	1.76 ± 1.44	None
PHQ-9 Score 5 – 9	74	26.7	6.62 ±1.37	Mild
PHQ-9 Score 10 – 14	17	6.1	11.53 ± 1.51	Moderate
PHQ-9 Score 15 – 19	11	4.0	16.18 ± 1.08	Moderately Severe
PHQ-9 Score 20 – 27	1	0.4	23.00 ± -	Severe
Suicidal thoughts - Yes	9	3.2	13.33 ± 3.43	-
Suicidal thoughts - No	268	96.8	4.01 ± 3.87	-

To identify reliable predictors of the PHQ-9 score, three hierarchical linear regression models were constructed using the PHQ-9 score as the dependent variable (Table 4). Model 1 included demographic variables only, namely age and gender, and served as a baseline model to control for basic individual characteristics. In Model 2, we added workload-related and employment-related variables. These variables comprised flight hours during the past 12 months, perceived job security, and additional duties. The inclusion of these factors enabled a more differentiated assessment of the impact of occupational demands and employment conditions on the PHQ-9 score. Model 3 further extended the previous models by including variables related to working conditions. Specifically, psychosocial factors, environmental, and perceived on-board air quality factors were entered into the model. In addition, pilots’ self-rated performance was included. This final model enabled a more comprehensive evaluation of the contribution of working conditions and individual performance perceptions beyond demographic and employment-related influences.

**Table 4:** Predictors for the PHQ-9 score as linear regression models (N=277) Dependent variable: PHQ-9 score; * Significance p<.050; ** Significance p<.001 PHQ-9: Patient Health Questionnaire-9

Variable	Model 1: General demographic data			Model 2: Workload and job security			Model 3: Working conditions		
	B	Lower 95% CI	Upper 95% CI	B	Lower 95% CI	Upper 95% CI	B	Lower 95% CI	Upper 95% CI
Age	.012	-.037	.061	.025	-.026	.076	.029	-.016	-.074
Gender	-.668	-2.866	1.529	-.706	-2.837	1.426	-.509	-2.374	1.356
Flight hours 12 months	-	-	-	-.002	-.008	.004	.002	-.003	.008
Flight hours 12 months – quadr.	-	-	-	5,642 E-6	.000	.000	-3,806 E-7	.000	.000
Job security	-	-	-	2.070**	1.069	3.071	.763	-.163	1.690
Additional duties	-	-	-	-.640	-1.780	.501	-.706	-1.715	.303
Pilots’ performance	-	-	-	-	-	-	-.126	-.259	.008
Psychosocial factors	-	-	-	-	-	-	.681**	.472	.890
Environmental factors	-	-	-	-	-	-	.144*	.074	.241
Air quality on board	-	-	-	-	-	-	-.287	-.878	.304
R^2^ (corr.)	-.005			.072			.293		

In Model 1, neither age nor gender emerged as a significant predictor of the PHQ-9 score. In Model 2, flight hours and additional duties were not significantly associated with the PHQ-9 score, but participants with an insecure job had a significantly higher PHQ-9 score than those with a secure job (B=2.070; 95% CI: 1.069-3.071). Both models demonstrated poor explanatory power, with R² values of -.005 and .072, respectively.

When considering working conditions across its three dimensions and pilots’ performance in the analysis, the model's explanatory power improved, yielding an R² value of .293. In Model 3, both psychosocial and environmental factors were found to be significantly associated with the PHQ-9 score. The strongest association was observed for psychosocial factors, with a regression coefficient of B = .681 (95% CI: .472-.890, p < .001). Environmental factors also showed a significant association, with a regression coefficient of B = .144 (95% CI: .074-.304, p = .004). In contrast, air quality was not significantly associated with the PHQ-9 score. In Model 3, job security was not further significantly associated with the PHQ-9 score.

## Discussion

Our study assessed a sample of 277 airline pilots using the PHQ-9 score to screen for depressive disorders in order to identify predictors of pilots’ mental health. The study results indicated that poor working conditions, particularly psychosocial and environmental factors, were significantly associated with higher depressive symptoms. In contrast, no significant differences in depression severity were observed based on gender, age, flight hours, job security, additional responsibilities, or self-assessed performance of the pilots. The study results underscore the significance of psychosocial and environmental working conditions in explaining mental health disparities among pilots.

Mental health issues and suicidality of airline pilots form a big threat to the safety of air transportation. As a consequence, our study aimed to identify possible influencing factors in order to improve pilots’ mental health by reducing the prevalence of major depressive disorders. We found a prevalence of 10.4% (n=29) for at least moderate depressive disorders, which supports other studies [6]. In total, 3.2% (n=9) pilots in the current study admitted to having had suicidal thoughts within the last two weeks, which is also congruent with the findings of Wu et al. [6].

Unlike the study of Wu et al. [6], we did not oversample female pilots and were able to collect a sample that depicts the general gender distribution among airline pilots. In this study, female pilots showed a lower PHQ-9 score compared to male pilots, which, however, did not reach significance. This finding contradicts other studies, where female pilots score higher than male pilots [6], and is also contradictory to studies assessing depressive symptoms of the general population, where women consistently show a higher prevalence of depressive disorders [16]. This divergence may be due to our low number of female pilots, which could not serve as a representative sample.

As one of our major findings, pilots who had either lost their jobs or found themselves in an insecure employment situation showed a significantly higher PHQ-9 score compared to pilots in a secure employment situation. Since our data collection took place shortly after the widespread COVID-19-related worldwide pilot lay-offs, we were able to source a sufficient sample to state the sound assertion that job-loss or uncertainty are influential risk factors for showing symptoms of depressive disorders. Possible reasons include financial instability, a feeling of helplessness, and psychosocial stress, which can lead to chronic stress and anxiety, which has been shown to contribute to higher depression scores [17]. Accordingly, the association between job security and depressive symptoms was no longer significant when psychosocial working conditions were included in the regression model. Gorlich and Stadelmann have assessed levels of depression, anxiety, and stress in a sample of flight attendants before and during the COVID-19 pandemic and showed strong correlations between anxiety, stress, and depression scores [18].

To assess whether a high workload is related to depressive disorders, we focused on the flight hours during the last year as a measure of long-term workload. Previous studies of pilots or other professions showed a significant impact of workload on mental health [19]. In a sample of Spanish airline pilots, a high workload was associated with a higher level of fatigue and sleepiness [8], which could then smooth the way into mental disorders like depression, which was also proposed in a systematic review on flight attendants’ mental health [20]. In contradiction, our study did not find a significant association between flight hours and depression. Unlike in other studies [8], we did not use specific scales to measure long-term workload, but used the flight hours during the last 12 months as the only measure for long-term workload. Other factors like total duty time, number of flights per day, time-zone crossings, standby duties, or ground training events were not considered in this study, possibly explaining differences in the mental health of pilots in the literature and strongly limiting the power of this variable in our study to accurately predict the PHQ-9 score.

For the general working population, maintaining an optimal workload balance, avoiding both under-stimulation and burnout, was frequently identified as a key factor in promoting mental well-being, as it fosters a sense of accomplishment without inducing excessive stress or fatigue [21,22]. It remains unclear whether this general statement can also be applied to airline pilots. To evaluate the potential of workload in its various dimensions as a predictor of the PHQ-9 score, the utilization of specific scales could enhance the accuracy of the assessment, which could be subject to future research.

The most influential factors identified in the current study were psychosocial and environmental aspects of working conditions, both of which turned out to be reliable predictors for the PHQ-9 score, where psychosocial factors showed the highest regression coefficient. The study questionnaire included several sub-dimensions of psychosocial factors: interest in work, too much work, influence on working conditions, support of fellow-workers, and fear of change, where pilots mostly complained about a lack of influence and fear of change. Missing influence on working conditions may induce the feeling of helplessness, and worries about an upcoming change in the personal situation may contribute to a higher level of chronic stress and anxiety [8,18], which we saw to be correlated to higher levels of depression scores.

Environmental factors (e.g., noise, odour, stuffy air, too high or low temperature, draught air, light reflections) also showed a significant association with the PHQ-9 score. To the best of our knowledge, no study assessed the relationship between environmental factors at work and pilots’ mental health so far. Our results are supported by a meta-analysis of seafarers’ mental health, which found that environmental factors like air pollution or high or low ambient temperatures led to a higher level of mood disorders or anxiety [23]. Studies of a general working population showed that factors such as noise, lighting, and ergonomic issues can contribute to discomfort and stress, negatively impacting mental well-being at the workplace [24]. Also, when assessing the general population, several studies found a correlation between air pollution and increased levels of depression and other mental health issues [25,26].

In conclusion, all factors mentioned above, which demonstrated a notable impact on the PHQ-9 score, collectively contribute to elevated chronic stress and anxiety levels, which are regarded as a substantial risk factor for the emergence of depressive disorders.

Practical implications

The findings of the current study offer valuable insights for the prevention and promotion of pilots' mental health by targeting the reduction of chronic stress and anxiety levels. However, it is not possible to draw causal conclusions solely based on this due to the cross-sectional study design. Given the importance of this topic and the close link between pilots' mental health and flight safety [7], it would be worthwhile considering changes to pilots' working conditions, even if today's data may not provide solid statistical evidence. It is imperative that any modifications implemented are subject to rigorous scientific monitoring and utilisation for interventional studies, with the objective of quantifying the impact of specific interventions.

The measures presented below are merely suggestions and cannot be statistically substantiated by the findings of the study.

In order to enhance job security, a critical element in mental well-being, a multifaceted approach involving a combination of measures might be recommended. This might include guaranteeing work hours, providing long-term contracts, and implementing responsible and forward-looking management policies. In cases where pilot layoffs are unavoidable, mitigating the negative impact on mental health could be achieved through responsible strategies, including the application of social criteria, voluntary redundancy options, or severance packages. 

To mitigate the adverse effects of environmental factors on mental health, utilizing the most modern aircraft fleet could be advisable. However, it is understandable from an airline's perspective that this is not always feasible. Nonetheless, operators are legally required to ensure that aircraft are technically well-maintained and fully operational [27]. A personalized approach may be required for airlines to mitigate the negative impacts of environmental factors. This could include providing pilots with appropriate equipment, such as sufficient thermal clothing to protect against cold and drafts, and offering adequate noise reduction measures, such as high-quality headsets, particularly when operating older aircraft. Furthermore, measures improving air quality, like installing filters to reduce air pollutants, could be considered by the industry.

When examining psychosocial factors as the strongest predictors, it appears that pilots do not face issues related to a lack of interest or insufficient social support at work. Instead, they are more likely to experience excessive workloads, a lack of influence over their working conditions, and significant concerns about impending changes. The extent to which the pandemic has influenced the pilots' concerns remains unclear. However, enhancing pilots' perceived influence over their work environment presents a more complex challenge. Pilots typically operate according to rosters published well in advance. To increase their sense of influence, airlines could allow pilots to request specific days off or preferred routes, a practice already standard in many airlines [28]. Additionally, perceptions of influence over work standards and conditions may improve if pilots have opportunities to voice their proposals and concerns, potentially through the strengthening of pilot unions. Furthermore, employers should ensure a clear career development strategy for pilots. Clearly stated minimum qualifications and/or a fairly organized system for advanced positions (e.g., senior first officer, captain, training captain) could lead to a higher level of control and influence. In order to reduce the magnitude of work, airline management should ensure to recruit enough pilots ahead of time, and/or may improve the legal minimums of duty time requirements as a voluntary commitment. This could also help to achieve a more balanced workload and give pilots more chances of compensatory (physical) activity.

The implementation of generalized health promotion programs for obese pilots has been shown to be an effective tool to improve pilots’ mental health [29]. The meta-analysis conducted by Miguel et al., which focused on healthcare workers within the general working population, identified only modest effects of psychosocial interventions and e-health programs on mental health outcomes [30]. However, their study demonstrated a more substantial impact of physical activity-based interventions, particularly among employees in sedentary occupations. It is conceivable that similar benefits could apply to pilots as well.

Furthermore, pilots form a special group of employees, where mental health might be directly linked to a (at least) temporary license suspension. As a consequence, programs should be established to give pilots access to psychiatric counseling and treatment without risking their license. Wu et al. already explained in detail that professions such as pilots seem to have a higher propensity to seek psychological help when experiencing mental health issues [6]. Research of the general working population showed that a proactive screening for mental health issues and an employer-organized concierge service can improve the rate of employees accepting help for mental health issues [31]. Such a program could also be implemented within airlines; however, a prerequisite for this would be that pilots who are diagnosed with mental illnesses are protected from losing their jobs. Such a program could offer an interesting starting point for longitudinal studies.

Strengths and limitations

Our study was able to include a large and diverse sample of 277 participants, enhancing the generalizability of the findings. We assessed multiple aspects of working conditions and mental health, providing a holistic view of factors possibly associated with pilots' mental well-being. The study seems to address highly relevant issues in a timely context, since many pilots have been affected by the COVID-19 pandemic, facing unprecedented challenges. We used well-validated scores, such as the PHQ-9 score, to provide a high level of quantitative measures. The use of an anonymous online assessment tool could have been essential for pilots to participate in our survey.

Since we used an online questionnaire and collected self-reported data, we were not able to ensure that participants who did not meet the inclusion criteria would not participate in the survey. Moreover, it is not possible to fully guarantee the precision of the data due to the potential existence of multiple biases. The most significant potential bias is that of self-selection, in which the study's participants may have been individuals who, by virtue of their personal experiences, were predisposed to encountering mental health challenges. In addition, the phenomenon of a social-desirability bias, or recall bias, must be considered. This is particularly probable in contexts where sensitive data is collected, and randomisation of participants and the provision of a control group are not feasible. Furthermore, the high number of excluded datasets may have introduced selection bias if excluded pilots differed systematically from those retained, for example, in terms of mental health status, working conditions, or response patterns. We often faced the limitation of missing data, especially when dividing participants into groups. As a consequence, statistical limitations applied, where we could not use all statistical tools as desired.

The cross-sectional design of our study limits the ability to assess causality between variables; a longitudinal study would have been more effective in determining causative relationships. Our findings may not be generalized, since our sample may not be large enough, mainly concentrates on European and North American pilots, and may not depict pilots of other regions.

Future research directions

Further studies could include a longitudinal design to explore causality between workload, working conditions, and mental health over a time period. Such a design could help to further identify factors and trends that a cross-sectional design is not able to depict. For such studies, a specific questionnaire should be used to assess workload in order to find differences between flight hours and (long-term) workload in its different dimensions. A more diverse sample from more regions could help to understand how cultural and regional factors influence mental health.

Furthermore, interventional studies could be executed that monitor the impact of changes to pilots' working conditions, and the effect of stress management programs or mental health support services. Further studies may also include additional variables like the role of social support, inside and outside the workplace.

## Conclusions

This study found a significant prevalence of depressive disorders and suicidal thoughts among airline pilots and aimed to identify robust predictors of pilots' mental health. We identified job insecurity, exposure to adverse environmental factors, and suboptimal psychosocial working conditions as critical predictors of poor mental health among pilots. Our findings call for immediate action from airline management, unions, and regulatory bodies to address these issues, although the study design does not allow causal conclusions. Suggestions include offering job security and establishing programs to mitigate perceived impacts on working conditions, which might improve pilots' mental health and, in turn, the safety of airline transport. Additional measures like mental health screening and support programs without risking a pilot’s license may lower the threshold for early treatment of mental health disorders. Future research should focus on longitudinal studies to explore causality and the effectiveness of targeted interventions, with an emphasis on diverse and comprehensive sampling across different regions. Collaborative efforts are essential to enhance the mental well-being of pilots, ensuring safe and reliable air travel.
